# The multifaceted role of ATM protein in neural stem/progenitor cell biology and neurogenesis: beyond DNA damage response

**DOI:** 10.3389/fphar.2026.1751635

**Published:** 2026-04-15

**Authors:** Giulia Boni, Mariagrazia Grilli

**Affiliations:** Laboratory of Neuroplasticity, Department of Pharmaceutical Sciences, University of Piemonte Orientale, Novara, Italy

**Keywords:** ataxia-telangiectasia, ATM, DNA damage response, neural stem progenitor cells, neurodegeneration, NSPC

## Abstract

The Ataxia Telangiectasia Mutated (ATM) protein kinase is a well recognized master regulator of the DNA damage response (DDR) and cell cycle control whose dysfunction leads to the rare neurological disorder Ataxia Telangiectasia (AT). A mounting body of evidence has revealed unequivocally that ATM relevance extends far beyond its DDR role and includes critical non-canonical functions. This minireview summarizes the current knowledge on ATM role in neural stem progenitor cell (NSPC) biology and in neurogenesis. In particular, herein we highlight how ATM is crucial for NSPC proliferation, differentiation, and survival, acting not only as a guardian of genomic integrity but also as a key orchestrator of developmental timing. Furthermore, we discuss how ATM deficiency in AT leads to dysregulated NSPC proliferation, premature neuronal maturation, and impaired quality control during neurogenesis, potentially contributing to progressive neurodegeneration and complex neurological symptoms associated with this pediatric disorder. By integrating canonical and non-canonical mechanisms, this review may offer a more comprehensive understanding of ATM key role in maintaining brain homeostasis integrity from the stem cell level. Moreover, it adds a more complex perspective on AT pathogenesis and opens novel avenues for future therapeutic interventions.

## Introduction

Studying the function of the ATM (Ataxia Telangiectasia Mutated) protein is crucial for a better understanding of Ataxia Telangiectasia (AT), a rare pediatric neurodegenerative disorder. Since AT patients lack functional ATM, identifying parallel pathways or proteins that can partially substitute for ATM function or targeting the consequences of ATM deficiency, may potentially offer avenues for therapeutic interventions that can restore some protective functions or mitigate the progressive neurological deterioration characteristic of this devastating disease. This minireview specifically focuses on the current knowledge on ATM canonical and non-canonical functions in neural stem progenitor cell (NSPC) biology and in neurogenesis. Moreover, we discuss how ATM deficiency in AT leads to dysregulated NSPC proliferation, premature neuronal maturation, and impaired quality control during neurogenesis, potentially contributing to progressive neurodegeneration and complex neurological symptoms associated with this rare disorder.

### The kinase ATM: key structural features

**FIGURE 1:**
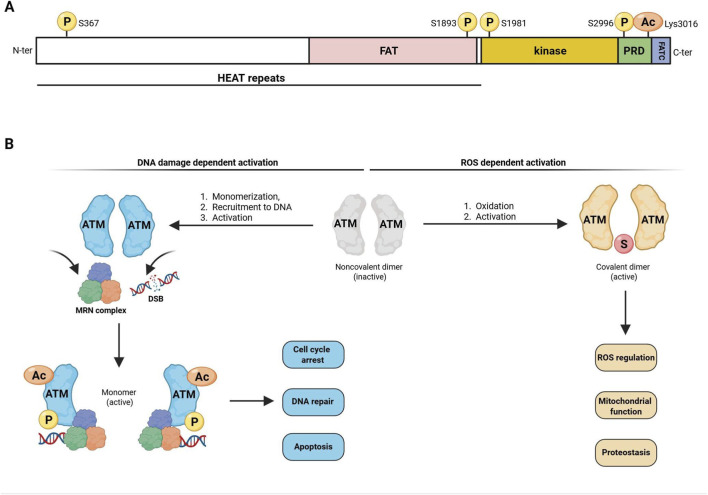
Structural features and activation mechanisms of ATM protein. **(A)** Schematic representation of ATM major domains: i) a FAT (FRAP, ATM and TRRAP proteins) domain which contains autophosphorylation sites crucial for substrate binding; ii) as a kinase, ATM has a serine/threonine kinase domain highly homologous to PI3K domain; iii) a PIKK regulatory domain (PRD); iv) a FATC domain essential for ATM full activation and interactions with substrates. Of note, autophosphorylation of Ser367, Ser 1893, Ser 1981, S2996 and acetylation of Lys3016 are important for ATM activation. ATM also contains α-helical HEAT repeat motifs which are critical for protein-protein and DNA interactions. **(B)** In its inactive state ATM is a noncovalent dimer (grey). ATM can be activated in a DNA damage-dependent manner (left): ATM is recruited to sites of double-strand DNA breaks (DSB) by the MRN complex (Rad50 in pink, MRE11 in blue, NBS1 in green), followed by ATM autophosphorylation (P) and acetylation (Ac). ATM/MRN complex association promotes ATM monomerization and its binding to downstream substrates involved in cell cycle arrest, DNA repair, or apoptosis. ATM can be also activated in a DNA- and MRN-independent manner by oxidative stress (right) through the formation of intermolecular disulfide bonds (S) converting the inactive monomer into an active covalent dimer which phosphorylates downstream targets involved in mitochondrial functions, ROS regulation, and proteostasis. Created with BioRender.

Ataxia Telangiectasia Mutated (ATM) is a serine/threonine protein kinase belonging to the family of phosphoinositide 3-kinase-related kinases (PIKKs) (Lovejoy and Cortez, 2009). At present, several hundreds of ATM substrates have been identified (Matsuoka et al., 2007). Over the past few years, the atomic structure of human ATM has been determined by high resolution cryo-electron microscopy (Baretić et al., 2017). Its N-terminal domain contains multiple α-helical HEAT-repeat motifs that are necessary for interacting with other proteins such as NBS1, p53 and BRCA1 (Fernandes et al., 2005). ATM also includes a FRAP-ATM-TRAPP (FAT) domain with autophosphorylation sites that are critical for substrate binding (Phan and Rezaeian, 2021). ATM also possesses a catalytic phosphoinositide 3-kinase (PI3K)-like kinase domain, a PIKK regulatory domain (PRD) as well as a FAT C-terminal domain (FATC) which are essential for its full activation, interaction with substrates (Bosotti et al., 2000), and regulation of its kinase activity (Jiang et al., 2006) (Figure 1A). The canonical form of ATM activation depends on DNA damage. ATM homodimer is recruited to sites of DNA double-strand breaks (DSBs) by the MRN complex comprising MRE11, RAD50 and NBS1. This is followed by ATM autophosphorylation at Ser 1981 (Bakkenist and Kastan, 2003), Ser367, Ser 1893, Ser2996 and Thr 1885 (Kozlov et al., 2011). It is also dependent on the acetylation of Lys3016 by the acetyltransferase TIP60 (Sun et al., 2007). These modifications induce kinase monomerization and promote ATM monomer interaction with DNA and its substrates, thereby stimulating the DDR machinery (Paull, 2015). ATM can also be activated in a DNA-independent manner by chromatin changes, hypotonic cellular stress or oxidative stress without the need for the MRN complex (Phan and Rezaeian, 2021). Under those circumstances ATM monomers form intermolecular disulfide bonds to generate active dimers, which then phosphorylate downstream targets (Guo et al., 2010) (Figure 1B).

### ATM and the rare neurodegenerative disease Ataxia Telangiectasia

AT is a rare autosomal recessive multi-organ disease characterized by early childhood onset and causing life shortening. No current therapy exists for AT. All AT cases are caused by mutations in the *Atm* gene located on chromosome 11q22-23 (Jeong et al., 2014). As of today more than 400 *Atm* gene mutations have been identified. The classical, most severe form of AT, is characterized by absent ATM protein activity and clinically marked by progressive ataxia and oculocutaneous telangiectasia. The disease generally manifests around 12–18 months of age with gait unsteadiness. Ataxia gradually worsens and by the age of 10 affected children are usually unable to walk. Other clinical symptoms such as dysarthria, oculomotor apraxia, dysphagia, dystonia, tremors and myoclonus more gradually develop and worsen (Barlow et al., 1996). Most patients are cognitively intact in childhood, although progressive cognitive impairment has been reported over time (Hoche et al., 2014). Associated immunodeficiency exposes individuals with AT to recurrent infections, especially in their respiratory tract. Predisposition to cancer development is manifested as a tendency to develop lymphoreticular malignancies in childhood, while different types of carcinomas may appear at older ages (Anheim et al., 2012). Endocrine abnormalities causing retarded growth and insulin-resistant diabetes are also present (Nissenkorn et al., 2016). Systemic premature aging is associated with AT (Aguado et al., 2022). Life expectancy is generally limited to 20–30 years of age in classical AT (Petley et al., 2022). In milder forms, associated with *Atm* gene mutations that allow residual ATM protein activity, symptoms are less severe, with later onset and slower progression resulting in increased life expectancy (Jeong et al., 2014). The most prominent neuropathological alteration in AT is cerebellar atrophy (Xu et al., 1996; Taylor et al., 2005). *Postmortem* studies revealed a significant loss of Purkinje and granule cells in the cerebella of AT children (Tai et al., 2018). To some extent also neuronal loss in striatum and substantia nigra has been detected in AT brain (Choy and Watters, 2018).

### Neural stem progenitor cells in the postnatal and adult brain

**FIGURE 2:**
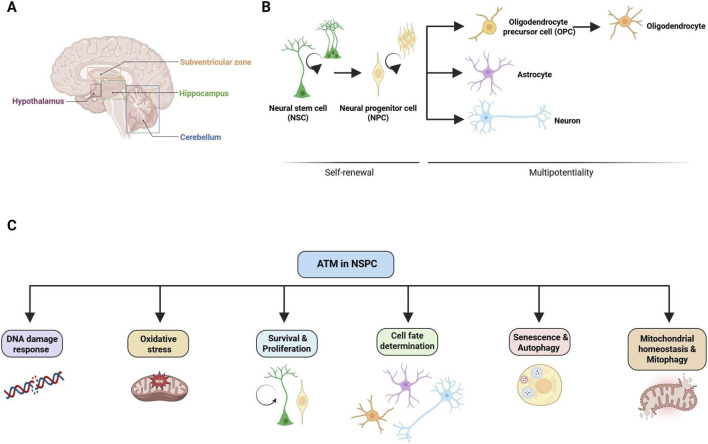
Adult neurogliogenesis and ATM functions in adult neural stem/progenitor cells (NSPC). **(A)** Major adult neurogliogenic niches in human brain are in the subventricular zone lining the lateral ventricles, the subgranular zone of the hippocampal dentate gyrus, the hypothalamus, and the cerebellum. **(B)** Schematic representation of key properties of neural stem cell (NSC) and neural progenitor cell (NPC): self-renewal and multipotency. In response to appropriate stimuli, NSCs which reside in a quiescent state start proliferating and can generate identical daughter cells to maintain the NSC pool and/or NPCs which are also proliferative cells. NPCs are multipotent cells which can undergo fate specification along both neuronal and glial lineages, giving rise to neurons, astrocytes and oligodendrocytes, derived from OPC. **(C)** Schematic representation of key ATM functions in NSPC. ATM is involved in several cellular processes that impact the homeostasis of NSPC: DNA damage response (DDR), oxidative stress response, survival/proliferation, differentiation, senescence/autophagy, and mitochondrial homeostasis/mitophagy. Created with BioRender.

Within the Central Nervous System (CNS), NSPCs represent a population of undifferentiated cells endowed with two key properties: self-renewal and multipotency. Self-renewal is the capacity to generate identical daughter cells, ensuring the maintenance of the stem cell pool, while multipotency refers to NSPC ability to differentiate into neurons, astrocytes and oligodendrocytes (Kriegstein and Alvarez-Buylla, 2009) (Figure 2B). Neurogenesis, the process by which neurons are generated from NSPCs, occurs during brain development but also postnatally and in adulthood. In the adult mammalian brain, neurogenesis is restricted to specialized microenvironments referred to as “niches”: the subventricular zone (SVZ) lining the lateral ventricles, the subgranular zone (SGZ) of the hippocampal dentate gyrus, and the hypothalamus (Kempermann et al., 2015; Sharif et al., 2021; Dumitru et al., 2025). The cerebellum has recently been described as another region where adult neurogenesis occurs (Sánchez-Gomar et al., 2024) (Figure 2A). Within niches, NSPCs are in a quiescent state and get activated in response to appropriate intrinsic and extrinsic stimuli. As a result of activation, NSPCs undergo tightly regulated stages: proliferation, differentiation into specific neural lineages, migration to target areas, and functional integration into preexisting neural circuits. Postnatal neurogenesis is vital for key physiological brain functions, including learning and memory, and response to stress (Deng et al., 2010). Importantly, its dysregulation is increasingly implicated in the pathogenesis of various neurodegenerative and neuropsychiatric diseases (Apple et al., 2017). Adult neurogenesis and NSPCs can also be pharmacologically modulated (Valente et al., 2015; Cuccurazzu et al., 2013; Bortolotto et al., 2019). For these reasons, studying neurogenesis and NSPCs represents a valuable approach for disclosing cellular and molecular pathophysiological mechanisms in several undertreated CNS disorders and for identifying novel therapeutic targets and avenues (Zhao and Moore, 2018).

### Direct impact of ATM on NSPC proliferation, differentiation, and survival

ATM plays a direct role in the regulation of NSPC behavior, influencing their proliferation, differentiation, and survival (Figure 2C). Such involvement extends beyond its canonical DDR function, indicating a specialized ATM role in neurogenesis in adulthood but also during development. ATM is abundantly expressed within dividing NSPCs (Allen et al., 2001); conversely its expression is markedly downregulated as these cells commit to differentiation. This expression pattern suggests a specific requirement for ATM during the active proliferative and early differentiation stages of neurogenesis and implies a tightly regulated role in the transition from stem cell to mature neuron (Allen et al., 2001). By taking advantage of ATM^−/−^ mice, a group of researchers suggested that ATM may serve as a key regulator for NSPCs by maintaining stemness and preventing senescence (Dong et al., 2022). They confirmed that ATM deficiency resulted in accumulation of DNA damage and accelerated senescence in SVZ NSPCs. Within 1 month, ATM deficient mice showed abnormally increased NSPC proliferation, whereas after 3 months NSPC proliferation significantly decreased resulting in the depletion of the NSPC pool over time. ATM is also crucial for cell fate determination, as described both *in vitro* and *in vivo*. Using ATM^−/−^ mice, Allen and colleagues studied ATM role in maintaining dentate gyrus NSPC genomic stability, cell fate specification and neuronal survival (Allen et al., 2001). They observed that in absence of ATM, hippocampal NSPCs show abnormally higher proliferationrates. Moreover, compared to *wild type* (WT) mice, ATM^−/−^ animals showed a lower increase in the number of BrdU^+^ NeuN^+^ neurons in response to running, an environmental stimulus that promotes NSPC proliferation, survival, and differentiation along the neuronal lineage. *In vitro* studies also confirmed that ATM^−/−^ NPCs were unable to properly differentiate towards MAP-2^+^ neurons but also RIP^+^ oligodendrocytes, while their ability to generate GFAP^+^ astrocytes was unaffected. Altogether data in ATM^−/−^ mice suggest that uncontrolled NSPC proliferation occurring when ATM is absent leads to exhaustion or deregulated differentiation of the stem cell pool and, in turn, this may potentially contribute to neuronal loss in AT. Carlessi and colleagues used an immortalized multipotent human neural stem-cell line to evaluate outcomes of ATM knockdown (KD) via shRNA (Carlessi et al., 2009). They found that exposure to ionizing radiation (IR)-induced DNA lesions was associated with phosphorylation of ATM and p53. ATM KD attenuated differentiation-associated apoptosis and response to IR, but had no effect on NSC proliferation, self-renewal and genomic stability. Compared to WT cells, ATM deficient NSCs generated similar numbers of MAP-2 or βIII-tubulin immunoreactive neurons; however, the yield of GABAergic neurons was reduced by 50%. Moreover, ATM KD attenuated the yield of Gal-C^+^ and CNPase^+^ oligodendrocytes. These results suggest that ATM deficiency does not impair overall neurogenesis, but its presence potentially guides NSPCs towards their correct neuronal identity and ensures their viability throughout the developmental process. Furthermore, they suggest that ATM deficiency also affects glial cells to some extent. At the molecular level, ATM orchestrates proliferation and neuronal differentiation of NSPCs through the phosphorylation of target proteins, such as 53BP1, primarily known for its role in DDR (Mirza-Aghazadeh-Attari et al., 2019). Recently, some researchers demonstrated that 53BP1 also supports NSPC stemness maintenance (Sunatani et al., 2024). They showed that hNSCs with depleted 53BP1 exhibit reduced self-renewal ability compared with control cells, as revealed by decreased neurosphere size and increased differentiation into neural or glial cells. Other studies confirmed that 53BP1 plays a crucial role in NSPC pool maintenance (Lim et al., 2024). They showed that ATM-dependent phosphorylation of 53BP1 at serine 25 is required for NSPC proliferation, neuronal differentiation and maturation in cortical brain organoids. The study suggested that lack of ATM-mediated phosphorylation of 53BP1 leads to earlier activation of neurodevelopmental genes in ATM KO organoids, resulting in premature neuronal maturation and formation of smaller, disorganized cortical organoids. Altogether, these data identify ATM-mediated 53BP1 phosphorylation as an orchestrator which ensures properly timed stem cell differentiation and highlight the non-canonical role of ATM in regulating the timing of developmental gene expression during cortical development.

### Canonical role of ATM in DNA repair: relevance in NSPC

ATM plays an essential role in DDR and in the maintenance of genomic stability, indeed ATM deficiency leads to accumulation of DNA damage and decreased DDR in NSPCs (Dong et al., 2022). In the SVZ of three-month-old ATM^−/−^ mice, the number of γH2AX foci, a marker for DSBs, was increased compared to WT mice. Moreover, the expression of Rad51, Brca1 and Ku70 – which are pivotal for the homologous recombination (HR) and the nonhomologous end joining (NHEJ) repair pathways–was decreased in ATM^−/−^ NPCs. Interestingly, the capacity of hNSCs to activate DDR in response to irradiation, which induces DSBs and ATM signaling, was also investigated. After IR stimulation, Chk2, KAP1, p53 and SMC1 are phosphorylated in WT but not in AT NSPCs and accumulation of cleaved poly (ADP ribose) polymerase (PARP), a marker of apoptosis, is detected in WT but not in AT NSCs. This suggested that ATM deficiency confers radio-resistance in proliferative cells (Carlessi et al., 2013).

### Interplay of non-canonical ATM functions with NSPC biology and functions

Beyond its consolidated role in DDR and genome stability, ATM is emerging as a multifaceted regulator involved in diverse molecular processes that significantly impact neural cell homeostasis (Figure 2C). These non-canonical roles are increasingly recognized as critical contributors to the complex pathology of AT. Yet, more limited knowledge is available about the relevance of non-canonical ATM functions in NSPCs compared to other cell types. Here we summarize ATM deficiency impact on NSPCs with a specific focus on some of these non-canonical functions.

### ATM and oxidative stress in NSPC

ATM can be directly activated by oxidative stress, a process independent of DNA DSBs (Qiu and Huang, 2021). High levels of reactive oxygen species (ROS) have been widely discussed as contributors to neurodegeneration in AT (Lee and Paull, 2021). Literature reports confirm that both cells derived from AT patients and ATM-deficient mice exhibit higher ROS levels. Specifically, ATM^−/−^ mice exhibit higher ROS levels compared to WT mice, especially within the cerebellum (Kamsler et al., 2001). Furthermore, NOX-4 and 4-HNE, markers of oxidation, are significantly higher in AT NSPCs; conversely, SOD1, an antioxidant enzyme, is significantly reduced in AT compared to WT NSPCs (Sunderland et al., 2020). Elevated ROS levels are well known to negatively influence NSC proliferation and neurogenesis (Hou et al., 2012). Accordingly, ATM loss impairs proliferation of postnatal NSPCs through oxidative stress mediated p38 MAPK signaling as suggested by Kim and colleagues (Kim and Wong, 2009). They investigated the effects of oxidative stress on NSC primary cultures isolated from the SVZ of ATM^−/−^ mice. These cells showed impaired proliferation rate as well as increased ROS levels compared to the WT counterpart. In addition, increasing ROS levels significantly reduced ATM^+/+^ NSC proliferation. In ATM^−/−^ NSCs, Akt and Erk1/2 pathways resulted disrupted, together with enhanced activity of p38 MAPK. Treatment of ATM^−/−^ NSCs with the antioxidant N-acetyl-L-cysteine (NAC) or with a p38 MAPK inhibitor restored proliferation rate and reduced expression of p21 and p27. These findings suggest that ATM regulates NSC proliferation via activation of Akt and Erk1/2 pathways and suppression of ROS-p38 MAPK signaling. Recently, Sunatani and colleagues demonstrated that 53BP1 depletion in hNSCs resulted in significantly increased cellular ROS levels, accompanied by mitochondrial abnormalities and reduced proliferation (Sunatani et al., 2024). Interestingly, the reduced self-renewal ability and elevated ROS levels in 53BP1-deficient NSCs were restored with NAC treatment. Altogether these findings identify ATM as a central player in maintaining redox balance, a role of fundamental importance for highly metabolically active cells such as NSPCs. Moreover, they corroborate the idea that oxidative stress in NSPCs may contribute to neurodegeneration in AT.

### ATM role in mitochondrial homeostasis and mitophagy in NSPC

Mitochondria are crucial for cellular metabolism, stress response and homeostasis maintenance. Changes in the number, size, morphology and localization of mitochondria, known as mitochondrial dynamics, are fundamental for proper cellular functions. An imbalance in these processes can lead to dysfunctional mitochondria, a condition present in various neurodegenerative disorders (Chen et al., 2023). In recent years, ATM, also localized in mitochondria (Subramanian et al., 2022), has emerged as a key master regulator of mitochondrial homeostasis (Leeson et al., 2024). Studies in ATM-null mice have also suggested that AT should be considered, at least in part, as a mitochondrial disease (Valentin-Vega et al., 2012). Leeson and colleagues discovered that despite no significant difference in mitochondrial content, mitochondrial membrane potential is significantly reduced in AT NSPCs compared to their WT counterpart (Leeson et al., 2024). A recent study also suggested that mitochondrial mass, estimated by mitochondrial outer membrane translocase TOM20 expression levels, is significantly higher in AT compared to WT NPCs. Moreover, BNIP3, a mitophagy inducer, is less expressed in AT compared to WT NSCs, suggesting that also mitophagy could be altered in AT (Sunderland et al., 2020). Altogether, these data not only confirm the role of ATM as a key regulator of mitochondrial homeostasis, but they also suggest that mitochondrial impairment in ATM deficient NPCs is not merely a downstream consequence of accumulating nuclear DNA damage (Chen et al., 2023), but that different signalling pathways are implicated.

### ATM role in NSPC senescence and autophagy

Senescence is the result of cellular stress factors or DNA damage, which cause permanent growth arrest (Ben-Porath and Weinberg, 2004). A recent study found that ATM deficiency results in accelerated NSPC senescence, as determined by increased SA-β-gal staining in the SVZ of three-month-old ATM^−/−^ mice compared to their WT counterpart (Dong et al., 2022). The study demonstrated that ATM deficiency leads to a significant increase in the expression of p21 and p53, markers of senescence, as well as a decrease of BMI1, a protein necessary for efficient self-renewal. ATM-deficient NSPCs derived from AT patients reprogrammed fibroblasts also exhibit senescence-like features (Sunderland et al., 2020). SIRT1, a NAD-dependent deacetylase, is significantly lower in AT NSPCs compared to WT counterpart; moreover, the levels of GATA4, a positive regulator of senescence-associated secretory phenotype (SASP), are increased in AT hNSPCs. Altogether these studies point not only to a role of ATM as key regulator of senescence in hNSPCs, but they also suggest that increased NSPC senescence may potentially contribute to neurodegeneration in AT. Under normal autophagic flux, protein aggregates formed by p62 are degraded by the autophagosome; however, when autophagy is impaired, p62 accumulates (Bjørkøy et al., 2005). Some researchers demonstrated that the expression of p62 is higher in AT hNSPCs compared to their WT counterpart, suggesting increased autophagy associated with ATM deficiency (Sunderland et al., 2020).

## Conclusion

ATM has been historically known for its key role in DDR and cell cycle control. Yet, in more recent years, in NSPCs, ATM is emerging as a multifaceted regulator with additional “non-canonical” functions, beyond DDR. These diverse functions span critical cellular processes including oxidative stress response, mitochondrial function, autophagy and cellular senescence. Literature data discussed in this review underscore that these non-canonical functions are also critical within the context of NSPC biology and neurogenesis, a relatively unexplored area of research. Experimental data demonstrate that ATM directly impacts NSPC proliferation, differentiation and survival, acting not only as a guardian of genomic integrity but also as a key orchestrator of their developmental timing. ATM deficiency, like in AT, can lead to deregulated NSPC proliferation, premature neuronal maturation, and impaired quality control mechanisms during neurogenesis. Deciphering ATM functions/dysfunctions in NSPCs can help researchers to better understand the pathogenesis of AT. The progressive neurodegeneration and complex neurological symptoms observed in AT could also be interpreted, at least in part, as a direct consequence of a multifaceted ATM dysfunction, which affects directly NSPCs and also their progeny, contributing to neuronal loss and functional decline. Altogether such evidence helps reframing AT as a disorder with also neurodevelopmental origin, where early developmental missteps lay the ground for postnatal progressive neurodegeneration. Furthermore, by moving beyond DDR, a more comprehensive and actionable understanding of ATM key role in maintaining brain health from the stem cell level could be achieved. Hopefully, these efforts may help paving the way for novel strategies of therapeutic intervention.
